# Salience and tolerance are not indicators of problematic social media use: Evidence from the Social Media Disorder Scale and the Bergen Social Media Addiction Scale

**DOI:** 10.1556/2006.2025.00073

**Published:** 2025-09-03

**Authors:** Víctor Ciudad-Fernández, Loïs Fournier, Tamara Escrivá-Martínez, Rosa Baños, Alfredo Zarco-Alpuente, Joël Billieux

**Affiliations:** 1 Department of Personality, Evaluation, and Psychological Treatments, University of Valencia, Valencia, Spain; 2 Polibienestar Institute, University of Valencia, Valencia, Spain; 3 Institute of Psychology, University of Lausanne, Lausanne, Switzerland; 4 CIBER Pathophysiology of Obesity and Nutrition (CIBEROBN), Carlos III Health Institute, Madrid, Spain; 5 Department of Basic Psychology, University of Valencia, Valencia, Spain; 6 Center for Excessive Gambling, Addiction Medicine, Lausanne University Hospital (CHUV), Lausanne, Switzerland

**Keywords:** components model of addiction, confirmatory factor analysis, core and peripheral criteria, problematic social media use, structural equation modeling

## Abstract

**Background and aims:**

The components model of addiction outlines six criteria shared by all addictive disorders. This proposal has been widely applied to conceptualize behavioral addictions, including problematic social media use (PSMU). However, certain criteria can be defined as “core” (e.g., mood modification, relapse, withdrawal, conflict), reflecting problematic involvement, while others as “peripheral” (e.g., salience, tolerance), reflecting non-problematic involvement. We evaluated whether a two-factor model distinguishing between core and peripheral criteria provides a better fit than the unifactorial model in PSMU. Additionally, we examined whether core and peripheral criteria exhibit different patterns of association with psychological measures.

**Methods:**

A total of 2,761 adolescents (*M* = 14.80 years, *SD* = 1.91 years) completed the Bergen Social Media Addiction Scale (BSMAS), the Social Media Disorder Scale (SMD), and measures of depression, anxiety, loneliness, life satisfaction, and self-esteem. Confirmatory factor analyses compared one-factor and two-factor models for the BSMAS and SMD. Associations were evaluated using structural equation models.

**Results:**

A two-factor model that distinguished core (i.e., mood modification, relapse, withdrawal, conflict) and peripheral (i.e., salience, tolerance) criteria provided a better fit than the unifactorial model for both scales. Core criteria were positively associated with depression, anxiety, and loneliness, and negatively associated with life satisfaction and self-esteem. Opposite patterns were observed for peripheral criteria: they were negatively associated with depression, anxiety, and loneliness, and positively associated with life satisfaction and self-esteem.

**Discussion and Conclusions:**

Our findings suggest that the components model of addiction may not be valid for assessing PSMU, promoting overdiagnosis and pathologization.

## Introduction

Since Isaac Marks introduced the term “non-chemical addictions” in 1990, this topic has gained significant traction, with excessive appetitive behaviors increasingly being recognized as genuine addictive disorders and labeled “behavioral addictions.” Notably, the American Psychological Association (APA) included *gambling disorder* in the “Substance-related and Addictive Disorders” section of the fifth edition of the *Diagnostic and Statistical Manual of Mental Disorders* (*DSM-5*; APA, 2013). More recently, in 2019, the World Health Organization (WHO) included both *gambling disorder* and *gaming disorder* in the “Disorders Due to Substance Use or Addictive Behaviors” section of the 11th edition of the *International Classification of Diseases* (*ICD-11*; Reed et al., 2022; WHO, 2019).

The paradigm shift that occurred when the APA aligned the first non-substance-related addictive disorder (i.e., gambling disorder) with substance use disorders in the *DSM-5* has been instrumental in the exponential trend of considering excessive appetitive behaviors as legitimate addictive behaviors. Substantial work conducted in the behavioral addiction field has followed what is commonly known as the “confirmatory approach” to behavioral addictions, which consists of recycling substance use disorder criteria to define and assess behavioral addictions (Billieux, Philippot, et al., 2015; Kardefelt-Winther et al., 2017; Van Rooij et al., 2018). An influential framework in the behavioral addiction field is the *components model of addiction* (Griffiths, 2005). This model is an adaptation of Brown's earlier work (1988, 1993, 1997) that conceptualizes addiction on the basis of a list of features (or symptoms) shared by all addictive disorders, whether substance-related or non-substance-related (Griffiths, 2005, 2017). The components model of addiction predates the formal recognition of gaming-related disorders in DSM-5 and ICD-11, and its six criteria show clear parallels with those diagnostic frameworks, especially the DSM-5 framework. For example, “relapse” maps onto “unsuccessful efforts to control or reduce use” (DSM-5 internet gaming disorder criterion 3) and “impaired control over gaming” (ICD-11 gaming disorder criterion A). Table 1 illustrates each component's definition and provides examples of problematic social media use items along with the corresponding (internet) gaming disorder DSM-5 and ICD-11 criteria.

**Table 1.** Components definition and items examples of main problematic social media use scales

Component	Definition of each component (Griffiths, 2005)	Example items from the SMD (Boer, van den Eijnden, et al., 2022; Van Den Eijnden et al., 2016)	Example items from the BSMAS (Andreassen, Torsheim, Brunborg, & Pallesen, 2012; Andreassen et al., 2016)	Type of component (Billieux et al., 2019; Charlton & Danforth, 2007)	DSM-5 Analogue “internet gaming disorder”	ICD-11 Analogue “gaming disorder”
Salience	The behavior is the most important activity, dominating cognition, emotions, and behavior.	During the past year, have you regularly found that you cannot think of anything else but the moment that you will be able to use social media again?	How often during the last year have you spent a lot of time thinking about social media or planning how to use it?	Peripheral component	Criterion 1: Preoccupation	No direct analogue
Tolerance	The need to increase the amount of the behavior to achieve the effects previously obtained.	During the past year, have you regularly felt dissatisfied because you wanted to spend more time on social media?	How often during the last year have you felt an urge to use social media more and more?	Peripheral component	Criterion 3: Tolerance	No direct analogue
Mood modification	The modification of subjective experience as a consequence of performing the behavior (e.g., arousing, tranquilizing, or allowing escape). Includes the euphoria experienced when performing or anticipating the behavior.	During the past year, have you often used social media to escape from negative feelings?	How often during the last year have you used social media in order to forget about personal problems?	Core/Peripheral component	Criterion 8: Use to relieve negative mood	No direct analogue
Relapse	The tendency to revert to earlier patterns of the behavior, with even the most extreme patterns quickly re-emerging after periods of abstinence or control.	During the past year, have you tried to spend less time on social media, but failed?	How often during the last year have you tried to cut down on the use of social media without success?	Core component	Criterion 4: Unsuccessful attempts to control or decrease use	Impaired control over gaming
Withdrawal	Unpleasant physical or emotional states occurring when the behavior is stopped or reduced.	During the past year, have you often felt bad when you could not use social media?	How often during the last year have you become restless or troubled if you have been prohibited from using social media?	Core component	Criterion 2: Withdrawal symptoms	No direct analogue
Conflict	Negative consequences arising in relationships with others or within oneself due to the behavior.	During the past year, have you had serious conflict with your parents, brother(s), or sister(s) because of your social media use?	How often during the last year have you used social media so much that it has had a negative impact on your job/studies?	Core component	Criterion 9: Jeopardization of significant relationship	Significant functional impairment

*Note*. In the initial outlines of the components model of addiction, the component “mood modification” was conceptualized under the name “euphoria” (Brown, 1988). The euphoria component is defined as the positive subjective experience people report because of engaging in a particular activity. In addition to positive subjective experiences, negative subjective experiences have been included in the mood modification component so that it can encompass positive or negative affective states related to one's involvement in an activity (Griffiths, 2005). Mood modification has been described either as a core or as a peripheral component in past research. Van Den Eijnden et al. (2016) first published both the original and short-form versions of the Social Media Disorder Scale (SMD). Boer, van den Eijnden, et al. (2022) later popularized the short form after a large cross-country study that examined its psychometric properties. Andreassen et al. (2012) first developed the Bergen Facebook Addiction Scale. Then, Andreassen et al. (2016) published the Bergen Social Media Addiction Scale (BSMAS), modifying the Bergen Facebook Addiction Scale by replacing the word “Facebook” with “social media.”

This model has been extensively cited and has stimulated the conceptualization of various behaviors and daily activities in which people may be highly engaged (e.g., technology-mediated behaviors, hobbies, sports) as potentially addictive behaviors. This conceptualization has been accompanied by the development of a plethora of brief assessment tools, typically composed of six items, covering the addiction “components” (Chen et al., 2020). In this context, various tools have been developed to assess a tentative “addiction” to technological devices, specific online activities, or apps (Cataldo, Billieux, Esposito, & Corazza, 2022; Starcevic, Billieux, & Schimmenti, 2018).

Among these instruments, two are particularly prominent in assessing problematic social media use (PSMU). The first is the six-item Bergen Social Media Addiction Scale (BSMAS) (Andreassen et al., 2016), which is based on the six criteria proposed in the components model of addiction. As of August 24, 2025, it had garnered 2,425 citations according to Google Scholar, making it the most popular assessment tool for measuring PSMU. The second is the nine-item Social Media Disorder Scale (SMD) (Van Den Eijnden, Lemmens, & Valkenburg, 2016), which is based on the internet gaming disorder criteria proposed in the *DSM-5* – criteria that are themselves derived from the “Substance-related and Addictive Disorders” section. Nonetheless, there is substantial overlap between the DSM-5 gaming disorder framework and the components model of addiction. The SMD had received 1,323 citations as of August 24, 2025, according to Google Scholar. Table 1 shows examples of items from these two PSMU assessment scales.

However, reliance on the components model of addiction – and more largely on substance use disorder criteria – to define behavioral addictions has been heavily criticized in recent years, mainly for failing to distinguish elevated but healthy involvement in a specific activity from pathological involvement (Billieux, Schimmenti, et al., 2015, 2019; Bottel et al., 2023; Flayelle, Schimmenti, Starcevic, & Billieux, 2022; Mihordin, 2012; Razum & Huić, 2024). Moreover, research findings and clinicians opinion suggests that the tolerance and salience criteria present limited or absent clinical validity and utility, as well as poor prognosis value (Castro-Calvo et al., 2021; Razum, Baumgartner, & Glavak-Tkalić, 2023), to the point that they have not been retained as central to define the condition in the *ICD-11* (Billieux, Stein, Castro-Calvo, Higuchi, & King, 2021; Reed et al., 2022). In contrast, manifestations like conflict or relapse were suggested as more valid indicators of gaming disorder (Castro-Calvo et al., 2021). In fact, seminal work on problematic use of computers and video games conducted by Charlton (2002) and Charlton and Danforth (2007) resulted in differentiating the components of addiction (Brown, 1993; Griffiths, 2005) into those measuring mere “engagement” (i.e., peripheral criteria) and those measuring “addiction” (i.e., core criteria). Recently, such differentiation has been emphasized, suggesting that, whereas core criteria directly indicate pathological behavior, peripheral criteria do not necessarily do so (Billieux, Flayelle, Rumpf, & Stein, 2019; Infanti, Valls-Serrano, Perales, Vögele, & Billieux, 2023). Although first recognized in the context of gaming disorder, this theoretical distinction has been observed across various domains such as social media use, online pornography use, and binge-watching of TV series (Deleuze, Long, Liu, Maurage, & Billieux, 2018; Infanti et al., 2023; Manchiraju & Sadachar, 2018; Mylonopoulos & Theoharakis, 2020, 2021; Orosz, Vallerand, Bőthe, Tóth-Király, & Paskuj, 2016; Tóth-Király et al., 2019; Vera Cruz et al., 2024; Verseillié, Laconi, Castro-Calvo, & Chabrol, 2023).

This crucial distinction between core and peripheral components remains largely ignored in most behavioral addiction research, as all six components are often considered to reflect a unitary construct of “addiction.” Although this approach is highly pragmatic in research because a single composite score that reflects “addiction” facilitates interpretation, it restricts detailed analysis of individual components (Cataldo et al., 2022), inflates false-positive cases, and pathologizes elevated but healthy involvement in appetitive behaviors (Kardefelt-Winther et al., 2017; Nogueira-López, Rial-Boubeta, Guadix-García, Villanueva-Blasco, & Billieux, 2023).

### Current study

Among the various behavioral addictions, we have selected PSMU as a compelling example to illustrate the potential pitfalls of applying substance use disorder criteria to appetitive behaviors. PSMU is not officially recognized as a disorder in either DSM-5 or ICD-11. Nevertheless, it is considered a growing public health concern from a public health perspective (WHO, 2024) and even a candidate for a mental condition when associated with negative consequences and functional impairment (Brand et al., 2022). Therefore, it is necessary to evaluate which assessment approach is valid in this context. PSMU is characterized by excessive and uncontrolled use of social media associated with a negative impact on personal, professional, and social functioning (Cataldo et al., 2022). Against this background, our aim in the present study was to test the (ir)relevance of using the components model of addiction to assess PSMU. Given their popularity and wide usage in this research field, we chose BSMAS and SMD to measure PSMU.

Psychometric studies conducted on these two questionnaires have systematically replicated a one-factor latent structure, implying that all assessed criteria are supposed to reflect a unitary latent construct of PSMU (Boer, Stevens, Finkenauer, Koning, & van den Eijnden, 2022; Brailovskaia & Margraf, 2022). However, critically, few studies have deliberately assessed and compared different measurement models, which would constitute a necessary prerequisite to investigate whether the items of these scales comprise a mixture of “core” and “peripheral” criteria and are thus likely to pathologize social media use. Notable exceptions are the work conducted by Fournier et al. (2023, 2024), Peng and Liao (2023), Verseillié et al. (2023), and Burén, Nutley, Crisci, and Thorell (2023) on the BSMAS and the Bergen Facebook Addiction Scale. These authors showed that these scales do not cohere into a one-factor structure, but rather reflect a two-factor structure that includes the peripheral components of salience and tolerance as a distinct factor. Verseillié et al. (2023) went further by including mood modification as an additional peripheral criterion. They reported similar associations between core and peripheral criteria for stress, anxiety, and time spent using Facebook. However, both Fournier et al. (2023) and Peng and Liao (2023) noted that peripheral criteria are not associated with psychopathological symptoms, suggesting that this factor measures “engagement in” rather than “addiction to” social media. Nonetheless, to the best of our knowledge, only a similar approach has been applied to the SMD in Burén et al. (2023). Guided by the data, these authors propose a new factor structure based on a principal component analysis finding of a two-factor structure, with salience, tolerance, relapse, and withdrawal as the heavy involvement factor, and the remaining items as the negative consequences factor. They did not find differential associations with constructs of interest (e.g., psychosomatic problems, low self-concept, social problems, and poor quality of life).

Therefore, in the present study, we aimed to explore further whether measurement instruments based on the components model of addiction that assess PSMU indeed mix core and peripheral criteria, using data collected from the two most popular PSMU scales: the six-item BSMAS (Andreassen et al., 2016) and the nine-item SMD (Van Den Eijnden et al., 2016). The research questions of this study are as follows:

1. RQ1: Does a one-factor or a two-factor model provide a more accurate representation of the latent structure underlying PSMU, as measured by the BSMAS and the SMD?
2. RQ2: How do core and peripheral criteria predict various measures of psychological well-being and distress?

## Methods

### Participants and procedure

Data were collected from 3,463 Spanish-speaking participants recruited from September 2023 to April 2024 in 16 educational centers located in Valencia and Madrid (Spain). Participation consisted of completing 14 psychometric instruments that assessed different psychological constructs (see “Secondary data study” folder for further details at the following link: https://osf.io/wc4ev/). They took an average of 35 min to complete the survey. No monetary compensation was provided; however, personalized reports were sent to all participating schools as an incentive. Each school received average scores for various mental health variables and PMSU, which were compared to the overall scores of the remaining participants. This approach ensured the anonymity of individual participants, and the identities of the participating schools were not disclosed.

Data collection was conducted in person by researchers and/or teachers, either via an online survey implemented on the *Qualtrics *platform (www.qualtrics.com) or paper, depending on the availability of technological devices. Paper responses were transcribed into *Qualtrics *by the principal investigator. Inclusion criteria were to be studying at a school in Spain (Valencia or Madrid) and to speak Spanish fluently. Exclusion criteria were not having access to social media (*n* = 88), being younger than 12 or older than 20 years (*n* = 45), not accepting participation (*n* = 318), or failing two or more of three attentional items out of a total of three (e.g., “If you are paying attention, check the option ‘Somewhat disagree’”) (*n* = 251). After applying the data exclusion criteria, the total sample comprised 2,761 participants (*M* = 14.80 years; *SD* = 1.91 years). Table 2 shows the sociodemographic data for the total sample.

**Table 2.** Sociodemographic data

Variable	*N* = 2,761^a^
**Course**	
1st year of compulsory secondary education	472 (17.0%)
2nd year of compulsory secondary education	468 (17.0%)
3rd year of compulsory secondary education	525 (19.0%)
4th year of compulsory secondary education	477 (17.0%)
1st year of baccalaureate	270 (9.8%)
2nd year of baccalaureate	227 (8.2%)
Intermediate vocational training	201 (7.3%)
Advanced vocational training	84 (3.0%)
Other	33 (1.2%)
**City**	
Valencia	2,487 (90.1%)
Madrid	274 (9.9%)
**Gender**	
Girl	1,345 (49.0%)
Boy	1,355 (49.0%)
Prefer not to answer	12 (0.4%)
Non-binary	3 (0.1%)
Other	37 (1.3%)
**Nationality**	
Spanish	2,408 (87.0%)
Other	347 (13.0%)
**Do you use social networks, such as *WhatsApp*, *TikTok*, *Instagram*, *Twitch*, *YouTube*, etc., more than three times a week?**	
Yes	2,674 (97.1%)
No	79 (2.9%)

*Note*. ^a^ Values are reported as *n* (%).

### Measures

*Bergen Social Media Addiction Scale (BSMAS) (Spanish version by Brailovskaia & Margraf, 2022; original English version by *Andreassen et al., 2016)

This scale is designed to measure PSMU. The BSMAS comprises six items, one for each criterion of the components model of addiction (e.g., tolerance: “You feel the need to use social media more and more”). It has traditionally been measured using a 5-point Likert scale. In the context of the present study, we also adapted it to a 6-point Likert scale for consistency and comparability with the SMD. Therefore, the item response options range from “strongly disagree” (1) to “strongly agree” (6). The composite score ranges from 6 to 36. The scale obtained a Cronbach's *α* of 0.85 in the Spanish validation and showed a negative association with mental health. Additionally, a recent meta-analysis found a positive association between the BSMAS and emotional symptoms (anxiety, stress, depression), as well as with internet gaming disorder symptoms (Bottaro, Griffiths, & Faraci, 2025). In the present sample, a ω of 0.90 and a Cronbach's *α* of 0.86 were obtained.

*Social Media Disorder Scale (SMD) (Spanish version by Boer, van den Eijnden, et al., 2022; original English version by Van Den Eijnden et al., 2016)*

This scale is designed to measure PSMU. The SMD consists of nine items that were developed explicitly from the internet gaming disorder criteria in *DSM-5*, which is directly derived from substance use disorder criteria (e.g., salience: “Have you regularly felt that you can't think of anything else but the moment when you can use social media again?”). Originally, it used a dichotomous scale (“Yes”/“No”) to measure each item. However, in accordance with the example of Savci, Ercengiz, and Aysan (2018), we transformed the dichotomous scale into a Likert scale. Specifically, we used a 6-point Likert scale for consistency and comparability with the BSMAS. Therefore, the item response options range from “strongly disagree” (1) to “strongly agree” (6). The composite score ranges from 6 to 36. The reported internal consistency on the original validation was a Cronbach's *α* of 0.81. In the Spanish validation, positive associations were found with psychosomatic complaints and greater intensity of online communication use, and a negative relationship was found with life satisfaction (Boer, van den Eijnden, et al., 2022). The global scale of our study achieved an ω of 0.92 and a Cronbach's *α* of 0.90.

*Patient Health Questionnaire-9 (PHQ-9) (Spanish version by Diez-Quevedo et al., 2001; original English version by Kroenke, Spitzer, & Williams, 2001)*

The adolescent version of this questionnaire was obtained from the AIDS Education & Training Center Program (www.aidsetc.org), adapted from the Patient Health Questionnaire Screeners website (www.phqscreeners.com). This scale measures symptoms of depression. This questionnaire features nine items in which higher scores indicate higher depression severity (e.g., “Have you felt little interest or found little pleasure in doing things?”). Items are rated from 0 (“not at all”) to 3 (“nearly every day”). Composite scores range from 0 to 27. The original validation study has shown an internal reliability coefficient of *α* = 0.89. This measure has shown a strong positive association with anxiety and depression measures (e.g., Beck's Depression Inventory), as well as a negative association with social and occupational functioning (Ferreira, Sousa, & Salgado, 2019; Rahman, Dhira, Sarker, & Mehareen, 2022). In this sample, the scale showed a McDonald's ω of 0.93 and a Cronbach's *α* of 0.91.

*Generalized Anxiety Disorder Scale* (*GAD-7) (Spanish version by *Crockett, Marcelo, Martínez, & Ordóñez-Carrasco, 2022*; original English version by Spitzer, Kroenke, Williams, & Löwe, 2006*)

The GAD-7 comprises seven items (e.g., “You have worried too much about different things”) that measure symptoms of anxiety. Items are on a 4-point scale ranging from 0 (“not at all”) to 3 (“nearly every day”). The composite score ranges from 0 to 24. The scale showed a Cronbach's *α* of 0.86 and a Spearman-Brown coefficient of 0.82 in the Spanish-speaking validation. This measure has shown a strong positive association with another anxiety scale (Pediatric Anxiety Rating Scale), as well as a moderate association with depression (Mossman et al., 2017; Seo & Park, 2015). This sample obtained a McDonald's ω of 0.94 and a Cronbach's *α* of 0.92.

*Three-Item Loneliness Scale* (*TILS) (Spanish version by* *Trucharte* et al.*, 2023**; original English version by Hughes, Waite, Hawkley, & Cacioppo, 2004*)

This scale measures loneliness and comprises three items (e.g., “How often do you feel you lack companionship?”). The response scale ranges from 1 (“hardly ever”) to 3 (“often”). The composite score ranges from 3 to 9. The Spanish validation study reported a Cronbach's *α* of 0.82. The TILS showed moderate positive associations with depression and anxiety, and a moderate negative association with well-being and neighborhood belonging (Trucharte et al., 2023). In this study, a McDonald's ω of 0.89 and a Cronbach's *α* of 0.88 were obtained.

*Satisfaction With Life Scale (SWLS-3) (Spanish version by* *Ortuño-Sierra, Aritio-Solana, Chocarro de Luis, Nalda, & Fonseca-Pedrero, 2019**; original English version by Diener, Emmons, Larsen, & Griffin, 1985)*

This questionnaire was used to assess life satisfaction. Originally composed of five items, Kjell and Diener (2021) proposed an adaptation, using only the first three of the original five items (e.g., “I am satisfied with my life”). The item response options range from “strongly disagree” (1) to “strongly agree” (7). The composite score on the scale ranges from 3 to 21. The Spanish validation obtained a McDonald's ω of 0.85. This same study found negative associations between this variable and emotional problems, peer problems, and hyperactivity, as well as a small but positive association with prosocial behavior (Ortuño-Sierra et al., 2019). In our sample, internal consistency reliability was similar (0.87 for both ω and *α*).

*Single Item Self-esteem* (*SISE) (Spanish version by* *Domínguez-Lara, 2020**; original English version by Robins, Hendin, & Trzesniewski, 2001*)

This scale measures self-esteem with only one item (i.e., “I have a high self-esteem”). The score ranges from 1 (“completely disagree”) to 5 (“completely agree”). The Spanish validation obtained a high correlation with the Rosenberg Self-Esteem Scale (ranging from 0.523 to 0.622). German validation obtained a correlation with the Rosenberg Self-Esteem Scale of 0.75 (Brailovskaia & Margraf, 2020), higher than the original validation (*r* = 0.51; Robins et al., 2001). The German validation also found positive associations between this construct and extraversion, openness, and conscientiousness, as well as negative associations with depression, narcissism, and neuroticism.

### Data analytic strategy

#### Missing data handling

First, missing values were handled. Of the participants, 15.86% did not respond to all items included in the survey. The percentage of missing data across items included in the complete survey ranged from 6.95% to 11.99%. Little's test indicated that the data were not missing completely at random (*p* < 0.05; Little, 1988). Second, the missRanger R package (Mayer & Mayer, 2019) was used to impute missing values by using random forests with the ranger algorithm, incorporating the predictive mean matching option (Wright & Ziegler, 2017). This package provides a faster imputation method than the alternative missForest package (Stekhoven & Bühlmann, 2012). In addition, including the predictive mean matching option enhances the plausibility of the imputed values (El Badisy, Graffeo, Khalis, & Giorgi, 2024). The imputation converged after six iterations with 1,000 trees in each iteration, resulting in a final mean out-of-bag error of 0.53. Notably, alternative missing data handling methods (i.e., listwise deletion) were also used; their corresponding results are available in the supplementary materials.

#### Confirmatory factor analyses

To address RQ1, we tested three factor structures for both the BSMAS and the SMD. Hence, six confirmatory factor analysis (CFA) models were fitted. In Model 1, we fitted the classical one-factor model reported in the original validation articles (Andreassen et al., 2016; Van Den Eijnden et al., 2016). Two alternative models were then fitted to examine the distinction between peripheral and core criteria. To account for a potential distinction between core and peripheral criteria (Billieux et al., 2019; Charlton & Danforth, 2007), we fitted Model 2, a two-factor solution that included salience, tolerance, and mood modification as peripheral factors, and relapse, withdrawal, and conflict as core factors. In Model 3, the same distinction was tested, but mood modification was included as a core rather than a peripheral criterion, in accordance with recent work conducted on the BSMAS (Fournier et al., 2023, 2024; Peng & Liao, 2023).

To fit the three models, we employed the robust maximum likelihood estimator due to its suitability for Likert scales with more than five response options (Finney & DiStefano, 2006). Model fit was evaluated by using the comparative fit index (CFI) and the Tucker-Lewis index (TLI), which were considered excellent above 0.95, as well as the root mean square error of approximation (RMSEA) and the standardized root mean square residual (SRMR), which were considered excellent at below 0.08 for the SRMR and below 0.06 for the RMSEA (Hu & Bentler, 1999). Model comparisons were assessed with the Akaike information criterion (AIC) and the Bayesian information criterion (BIC), with lower values indicating better fit. Furthermore, chi-square difference tests were used to compare nested models. The lavaan R package was used to compute CFA models (Rosseel, 2023). Lastly, internal consistency reliability was reported with Cronbach's *α* and McDonald's ω by using the R package semTools (Jorgensen, Pornprasertmanit, Schoemann, & Rosseel, 2022).

#### Structural equation analyses

To evaluate RQ2, we specified two Structural Equation Models (SEM), one for the BSMAS and one for the SMD. SEM was chosen for its ability to model multiple latent constructs simultaneously and to reduce measurement error. In each model, we treated all constructs as latent: core and peripheral PSMU criteria were modeled as exogenous factors, and life satisfaction, self-esteem, loneliness, depression, and anxiety were modeled as endogenous factors, following the best-fitting CFA structures for the BSMAS and SMD. Parameters were estimated using the WLSMV estimator, and model fit was evaluated according to the aforementioned cutoff criteria for the fit indices. This estimator was chosen due to its superiority to other estimators when treating categorical items (e.g., Robust Maximum Likelihood; Li, 2016). Consistent with the CFAs, the lavaan R package was used (Rosseel, 2023).

### Ethics

Ethical approval for this study was granted by the Ethics Committee of the University of Valencia (Spain) (procedure number: 2675827). All participants provided informed consent prior to their participation in the present study. A passive informed consent procedure was followed, whereby parents or legal guardians were informed of the study by each participating school. These data have already been used in a previous original research article (see https://osf.io/wc4ev/).

## Results

### Confirmatory factor analyses

Chi-square difference tests between all confirmatory analysis models suggested that the two-factor models (i.e., Model 2, Model 3) presented a significantly better fit than did the classic unifactorial model (i.e., Model 1) (*p* < 0.05). Furthermore, the fit indices were best for Model 3 regarding both the BSMAS and the SMD. Table 3 shows the fit indices for each model and the comparison among nested models. The RMSEA value is high for models 1 and 2 in SMD scale. In addition, the CFI and TLI values of the same models do not reach the cutoff value to be considered excellent (i.e., 0.95), while the fit indices are bust for Model 3. Conversely, the degree of misfit is lower in the BSMAS models. Consequently, for further analyses, we retained Model 3, with salience and tolerance reflecting a “peripheral criteria” factor, and mood modification, relapse, withdrawal, and conflict reflecting a “core criteria” factor. This was done independently for both the SMD and the BSMAS questionnaires.

**Table 3.** Confirmatory factor analysis models

Model	χ^2^	*df*	*p*-value	CFI	TLI	RMSEA	SRMR	AIC	BIC	Δχ^2^	Δ*df*	χ^2^ difference test *p*-value
SMD Model 1	530.070	27	<0.001	0.917	0.889	0.082	0.046	78,642.76	78,749.38	–	–	
SMD Model 2	511.755	26	<0.001	0.919	0.889	0.082	0.046	78,611.86	78,724.41	18.559	1	<0.001
SMD Model 3	449.495	26	<0.001	0.930	0.903	0.077	0.043	78,524.43	78,636.97	73.818	1	<0.001
BSMAS Model 1	155.859	9	<0.001	0.961	0.935	0.077	0.033	53,301.26	53,372.35	–	–	
BSMAS Model 2	108.127	8	<0.001	0.974	0.951	0.067	0.030	53,236.19	53,313.19	44.987	1	<0.001
BSMAS Model 3	44.999	8	<0.001	0.990	0.982	0.041	0.019	53,151.40	53,228.41	113.37	1	<0.001

*Note*. SMD Model 1: one-factor model; SMD Model 2: two-factor model (tolerance, salience, and mood modification as peripheral criteria); SMD Model 3: two-factor model (tolerance and salience as peripheral criteria); BSMAS Model 1: one-factor model; BSMAS Model 2: two-factor model (tolerance, salience, and mood modification as peripheral criteria); BSMAS Model 3: two-factor model (tolerance and salience as peripheral criteria). The second and third models were evaluated against their nested model (the first model). To compare these nested models, we used Satorra-Bentler scaled difference tests.

Table 4 presents composite reliability estimates. Overall, internal consistency is highest in Model 1 for the BSMAS and the SMD. Among the two-factor models, Model 3, which includes only tolerance and salience as peripheral criteria, shows higher reliability values than does Model 2, which also includes mood modification.

**Table 4.** Internal consistency reliability estimates

Model	Factor	Number of items	Cronbach's *α*	McDonald's ω
BSMAS Model 1	Factor 1	6	0.830	0.826
BSMAS Model 2	Factor 1	3	0.715	0.712
	Factor 2	3	0.738	0.755
BSMAS Model 3	Factor 1	4	0.757	0.757
	Factor 2	2	0.762	0.762
SMD Model 1	Factor 1	9	0.864	0.861
SMD Model 2	Factor 1	6	0.815	0.806
	Factor 2	3	0.686	0.715
SMD Model 3	Factor 1	7	0.832	0.825
	Factor 2	2	0.715	0.725

*Note*. BSMAS Model 1: one-factor model; BSMAS Model 2: two-factor model (tolerance, salience, and mood modification as peripheral criteria); BSMAS Model 3: two-factor model (tolerance and salience as peripheral criteria); SMD Model 1: one-factor model; SMD Model 2: two-factor model (tolerance, salience, and mood modification as peripheral criteria); SMD Model 3: two-factor model (tolerance and salience as peripheral criteria).

### Structural equation modeling

**Fig. 1.**
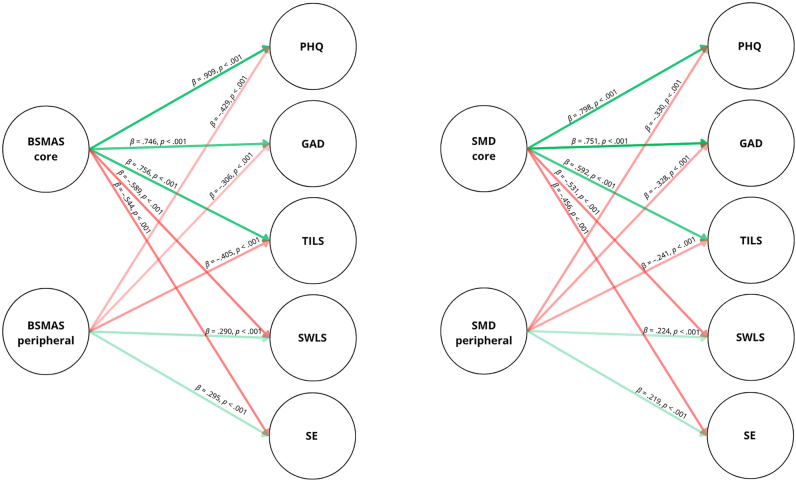
Standardized regression estimates for the two-factor SEM models of BSMAS and SMD. *Note*. For clarity, indicators, intercepts, covariances, thresholds, and unique variances are not depicted. To compute the SEM models, we selected the two-factor model for the two questionnaires (i.e., including only tolerance and salience as peripheral components). BSMAS = Bergen Social Media Addiction Scale; SMD = Social Media Disorder Scale; PHQ = Patient Health Questionnaire-9; GAD = Generalized Anxiety Disorder Scale-7; TILS = Three-Item Loneliness Scale; SWLS = Satisfaction With Life Scale; SE = Single Item Self-Esteem.

Lastly, SEM highlighted the relationships of core and peripheral criteria with depression, anxiety, loneliness, life satisfaction, and self-esteem. For both the BSMAS and the SMD, the peripheral criteria showed a negative relationship with depression, anxiety, and loneliness and a positive relationship with life satisfaction and self-esteem. In contrast, for both the BSMAS and the SMD, the core criteria showed a positive relationship with depression, anxiety, and loneliness and a negative relationship with life satisfaction and self-esteem. Figure 1 displays the SEM models for the BSMAS and the SMD along their standardized regression paths. Table 5 presents the factor loadings for each BSMAS and SMD item on their respective factors. All factor loadings exceed 0.550 and are statistically significant. Moreover, the factor loadings on the peripheral factors all exceeded 0.725. Lastly, Table 6 summarizes the fit indices of the two SEM models, indicating a good fit to the data according to the proposed cutoffs for the fit indices.

**Table 5.** Factor loadings for core and peripheral criteria in the BSMAS and SMD

Scale	Item	Factor	Factor loadings (λ)	*p*-value
BSMAS	1	Peripheral	0.725	–
	2	Peripheral	0.857	<0.001
	3	Core	0.777	–
	4	Core	0.569	<0.001
	5	Core	0.629	<0.001
	6	Core	0.650	<0.001
SMD	1	Peripheral	0.735	–
	2	Peripheral	0.771	<0.001
	3	Core	0.647	–
	4	Core	0.586	<0.001
	5	Core	0.584	<0.001
	6	Core	0.622	<0.001
	7	Core	0.602	<0.001
	8	Core	0.776	<0.001
	9	Core	0.658	<0.001

*Note*. Factor loadings for the first indicator of each factor were fixed to 1.00 for identification and are not tested. BSMAS = Bergen Social Media Addiction Scale, SMD = Social Media Disorder Scale.

**Table 6.** Fit indices for the two-factor SEM models of the BSMAS and the SMD

Model	χ^2^	*df*	*p*-value	CFI	TLI	RMSEA	SRMR
BSMAS	3,236.801	357	<0.001	0.942	0.934	0.054	0.040
SMD	3,508.567	444	<0.001	0.938	0.931	0.050	0.041

## Discussion

The components model of addiction (Brown, 1993; Griffiths, 2005) has been influential in the behavioral addiction field, being used to develop several scales conceptualizing a wide range of activities and common behaviors as tentative or emerging conditions. In this study, considering the high global prevalence and popularity of social media, we focused on PSMU to showcase the potential pitfalls of using substance use disorder criteria to conceptualize and assess behavioral addictions or excessive appetitive behaviors. To this end, we tested and challenged the assumed unidimensional latent structure of two popular scales that assess PSMU (i.e., the BSMAS and the SMD) on the basis of the components model of addiction and the *DSM-5* framework, which recycles substance use disorder criteria to define online addictive behaviors.

Our results showed that a two-factor structure (considering salience and tolerance as peripheral criteria and the remaining items as core criteria) shows a better adjustment to the data than does the widely used one-factor structure. Such results align with those reported in Fournier et al. (2023, 2024), who used four independent Italian samples that had completed the BSMAS. In addition, a two-factor structure that considered mood modification as a peripheral rather than a core criterion also outperformed the one-factor structure in PSMU (Model 2); yet, considering it as a core criterion yielded a better fit to the data (Model 3). Griffiths (2005) defines the mood modification criterion as the “subjective experience that people report as a consequence of engaging in the particular activity (i.e., they experience an arousing ‘buzz’ or a ‘high’ or paradoxically a tranquilizing and/or destressing feel of ‘escape’ or ‘numbing’).” However, most brief psychometric tools used to assess behavioral addictions overlook the positive reinforcement aspect of this definition, focusing solely on negative reinforcement. This is true for both the SMD and the BSMAS. Mood modification for positive reinforcement was proposed to be a peripheral criterion in the initial work conducted by Charlton (2002) and Charlton and Danforth (2007), whereas recent studies frequently suggest that mood modification for negative reinforcement constitutes a core criterion (Burén et al., 2023; Peng & Liao, 2023). Furthermore, avoiding emotional discomfort through problematic behaviors has been identified as a bridge symptom across other problematic behaviors (Li, Mu, Xie, & Kwok, 2023). Peng and Liao (2023) also identified a group of highly involved, but non-problematic, users from the BSMAS, characterized by high scores on salience and tolerance, but low scores on other criteria. This could account for why Model 3 (which considers mood modification a peripheral criterion) seems to provide a better fit than Model 2 (which treats mood modification as a core criterion).

SEM analysis indicated that core criteria positively predict depression, anxiety, and loneliness and negatively predict life satisfaction and self-esteem, whereas peripheral criteria showed the opposite pattern. When we took into account the relationship between core criteria and psychological indicators, an inverse and significant relationship emerged with all dependent variables considered. Meta-analytic literature indicates a positive association between PSMU and depression, anxiety, and loneliness and a negative one with self-esteem and life satisfaction (Akkaş & Turan, 2023; Huang, 2022; Shannon, Bush, Villeneuve, Hellemans, & Guimond, 2022). In parallel, some studies have reported no association between salience/tolerance and psychological distress (Fournier et al., 2023), while some even indicated a negative relationship (Guo et al., 2022; Peng & Liao, 2023; Zarate, Ball, Montag, Prokofieva, & Stavropoulos, 2022). Negative mental health variables are typically associated with PSMU because both types of criteria (i.e., peripheral and core) are generally conflated within a single construct. However, item-level analysis contributed to providing a different picture and calls for not conflating all criteria into a single latent factor that reflects PSMU. Therefore, taking into account prior studies and the present results, it might be relevant to avoid the term “peripheral criteria” (which is not a widely used nor necessarily accepted term) and instead speak about “engagement indicators” (reflecting intensive but not necessarily problematic use) when referring to manifestations akin to salience and tolerance in PSMU.

The pattern of results found in this study can be understood in light of the dualistic model of passion formulated in Vallerand et al. (2003) and Vallerand (2008) which posits two types of passion. The first type, “harmonious passion,” stems from voluntary internalization of the activity into one's identity, allowing individuals to choose to engage in what they enjoy in a way that is fully integrated and does not interfere with daily life tasks and duties. In contrast, “obsessive passion” arises from pressured internalization, leading to a compulsive and unregulated need to perform the activity and to negative consequences and functional impairment. Although harmonious passion (for various types of activities) has been linked to positive outcomes, such as increased well-being and performance, obsessive passion has been associated with adverse outcomes, including emotional distress and rumination (Curran, Hill, Appleton, Vallerand, & Standage, 2015). Therefore, highly involved but non-problematic social media users are likely driven by harmonious passion, whereas problematic users are more likely driven by obsessive passion (see Billieux et al., 2019, for a similar discussion in the context of gaming).

Overall, our results suggest that both the BSMAS and the SMD assess two distinct latent constructs and that their items should not be used to compute a global score of “addictive social media use.” Although this study focused on PSMU, the theoretical distinction between high involvement and pathological involvement is not exclusive to social media use: it has been observed in the context of various appetitive behaviors, such as exercising, binge-watching TV series, using online pornography, gaming, and gambling (Charlton & Danforth, 2007, 2010; Flayelle et al., 2022; Granziol et al., 2023; Katz et al., 2024; Vera Cruz et al., 2024; Whelan, Laato, Islam, & Billieux, 2021). For instance, exercise addiction scales were often conceptualized within a one-factor framework (Terry, Szabo, & Griffiths, 2004), but were later adapted into a two-factor framework, allowing investigators to distinguish “high involvement” from “addiction,” the latter showing stronger associations with psychopathological outcomes (Granziol et al., 2023).

### Limitations and future research

This study has several limitations that must be addressed to contextualize its findings. First, the sample comprises solely adolescents from schools in Spain, which may limit the generalizability of the results to other age groups, settings, countries, or languages. Second, using only two items to evaluate the peripheral factor is a relevant limitation (Eisinga, Grotenhuis, & Pelzer, 2013). Generally, a greater number of items leads to better measurement accuracy, and SEM literature typically recommends using at least three observed variables per latent variable to ensure robust solutions and reduce estimation errors (Emons, Sijtsma, & Meijer, 2007; Heck & Thomas, 2020; Kline, 2005). However, having a large sample size (*N* ≥ 1000) may mitigate this problem (Marsh, Hau, Balla, & Grayson, 1998). Future efforts to assess both core and peripheral features of PSMU should require scales with more items per factor. An additional advantage of this approach is that it would enhance the content validity of these scales, as scales with a very limited number of items tend to assess overly narrow constructs (see, e.g., Smith, McCarthy, & Anderson, 2000). For instance, the 27-item SMD could be used to assess each criterion with at least three items (Van Den Eijnden et al., 2016). Third, this study employed the two most widely used scales for assessing PSMU (i.e., the BSMAS and the SMD) to demonstrate the issues associated with mixing peripheral and core criteria. However, it would have been useful to include, for comparison purpose, instruments grounded in ICD-11 criteria which do not include peripheral criteria, such as the Assessment of Criteria for Specific Internet-use Disorders-11 (ACSID-11; Müller et al., 2022) and the Social Media Use Disorder Scale (SOMEDIS-A; Paschke, Austermann, & Thomasius, 2021). Last, this study has explored the distinction between core and peripheral criteria in PSMU. However, this distinction should not be limited to this specific behavior; it should also be evaluated across other appetitive behaviors conceptualized as “problematic” behaviors to gain a broader understanding of its applicability and accuracy.

## Conclusions

Our findings highlight significant limitations in the two most popular scales for assessing PSMU, which hold important implications for the field. The BSMAS and the SMD conflate core criteria and peripheral criteria, thus mixing items reflecting engagement with items reflecting addiction. The current approach to PSMU assessment contributes to (over)pathologizing the use of social media. Indeed, including peripheral criteria to assess excessive behaviors contributes to inflated prevalence rates (Cheng, Lau, Chan, & Luk, 2021; Nogueira-López et al., 2023). This phenomenon has also been elegantly illustrated by Satchell et al. (2021) in their satirical (but empirical) study in which 69% of participants were classified as being addicted to spending offline time with friends.

We advocate for the critical evaluation of core criteria in behavioral addiction research and recommend removing peripheral criteria from assessments, as their inclusion pathologizes intensive – but healthy – involvement in appetitive behaviors. We also propose to rename peripheral criteria as engagement indicators and invite clinicians and researchers to be aware of these issues and be more cautious about using “quick and short” screening tools that conflate problematic and non-problematic items to assess a construct of “behavioral addiction,” which is often rooted in criteria developed to assess substance use disorders. To provide a constructive way forward, we also suggest adopting instruments grounded in ICD-11 criteria, such as the ACSID-11, which assesses impaired control over the behavior and functional impairment in important areas of daily life as key indicators of PSMU.

## Supplementary material

## Data Availability

The code and data analyzed in the current study are publicly available in the “Secondary data study” folder at the following link: https://osf.io/wc4ev/.
