# Supplementary material for: Salience and tolerance are not indicators of problematic social media use: Evidence from the Social Media Disorder Scale and the Bergen Social Media Addiction Scale
Source: J Behav Addict. 2025 Sep 3;14(3):1380–93. doi: 10.1556/2006.2025.00073 (PMC12486260; doi:10.1556/2006.2025.00073)
Supplement: Supplementary file 1 [file jba-14-1380-s001.pdf]

Ciudad-Fernández V. et al.: Salience and tolerance are not indicators of problematic social media use: evidence from the Social Media Disorder Scale and the Bergen Social Media Addiction Scale

https://doi.org/10.1556/2006.2025.00073

SUPPLEMENTARY MATERIALS

Table S1. Means, standard deviations, and correlations

Variable	*M*	*SD*	1	2	3	4	5	6	7	8	9	10
1. BSMAS	14.97	7.27										
2. Core BSMAS	10.27	5.18	.97**									
3. Peripheral BSMAS	4.70	2.56	.88**	.74**								
4. SMD	19.83	10.09	.83**	.82**	.70**							
5. Core SMD	15.92	8.26	.82**	.82**	.67**	.99**						
6. Peripheral SMD	3.91	2.31	.72**	.67**	.68**	.84**	.75**					
7. PHQ	7.37	6.06	.49**	.51**	.37**	.52**	.52**	.38**				
8. GAD	5.89	5.20	.46**	.46**	.36**	.48**	.48**	.35**	.81**			
9. TILS	4.19	2.10	.47**	.48**	.37**	.50**	.50**	.39**	.63**	.60**		
10. SWLS	13.88	5.93	.22**	.19**	.22**	.24**	.22**	.23**	-.06**	-.02	.22**	
11. SE	3.43	1.18	-.24**	-.26**	-.15**	-.24**	-.25**	-.14**	-.49**	-.42**	-.45**	.45**

Note. M and *SD* are used to represent mean and standard deviation, respectively. *p < .05. **p < .01.

Figure S1. Missing percentage values for each variable.

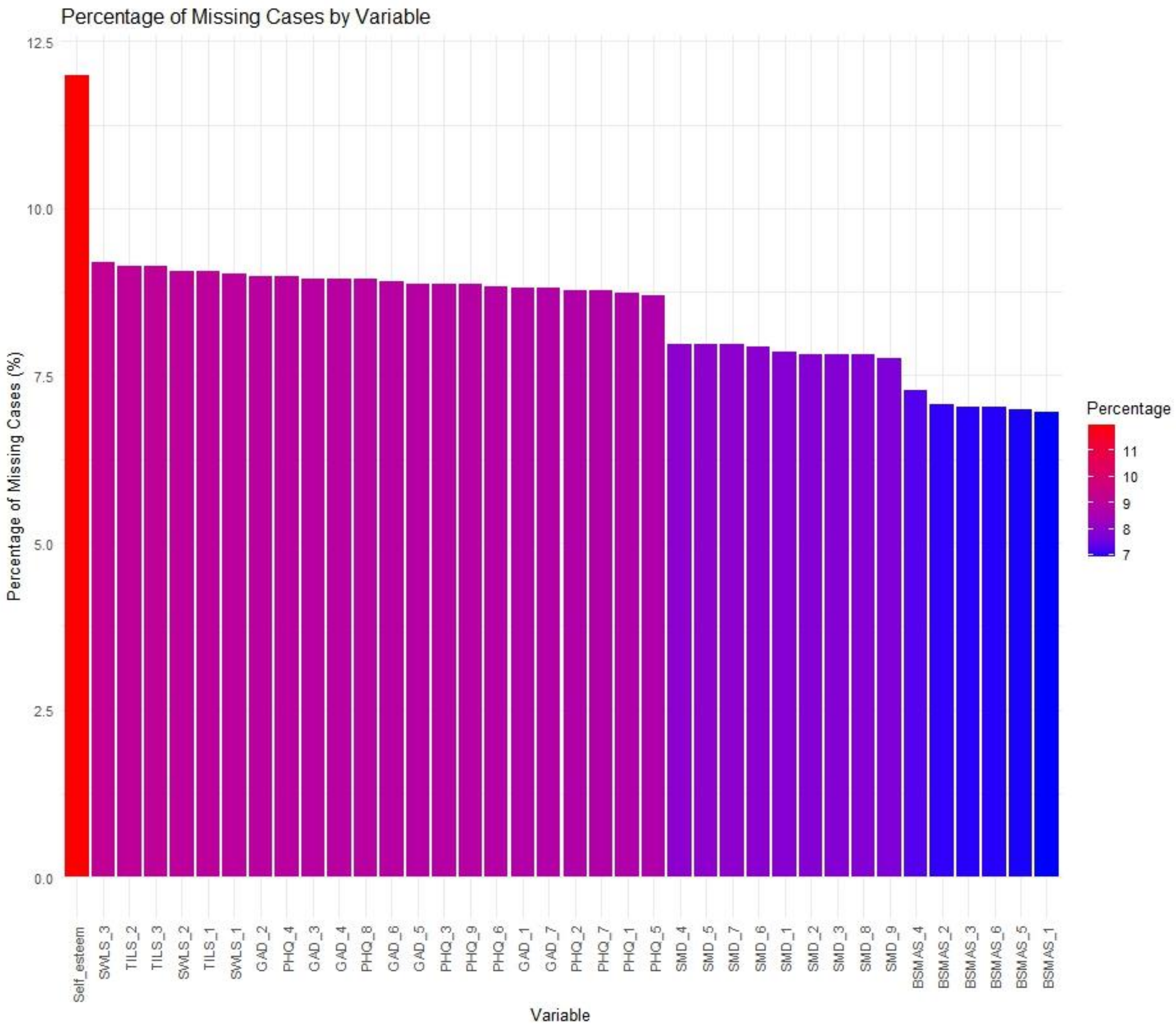

Figure S2. Distribution of the variables before and after the random forest imputation.

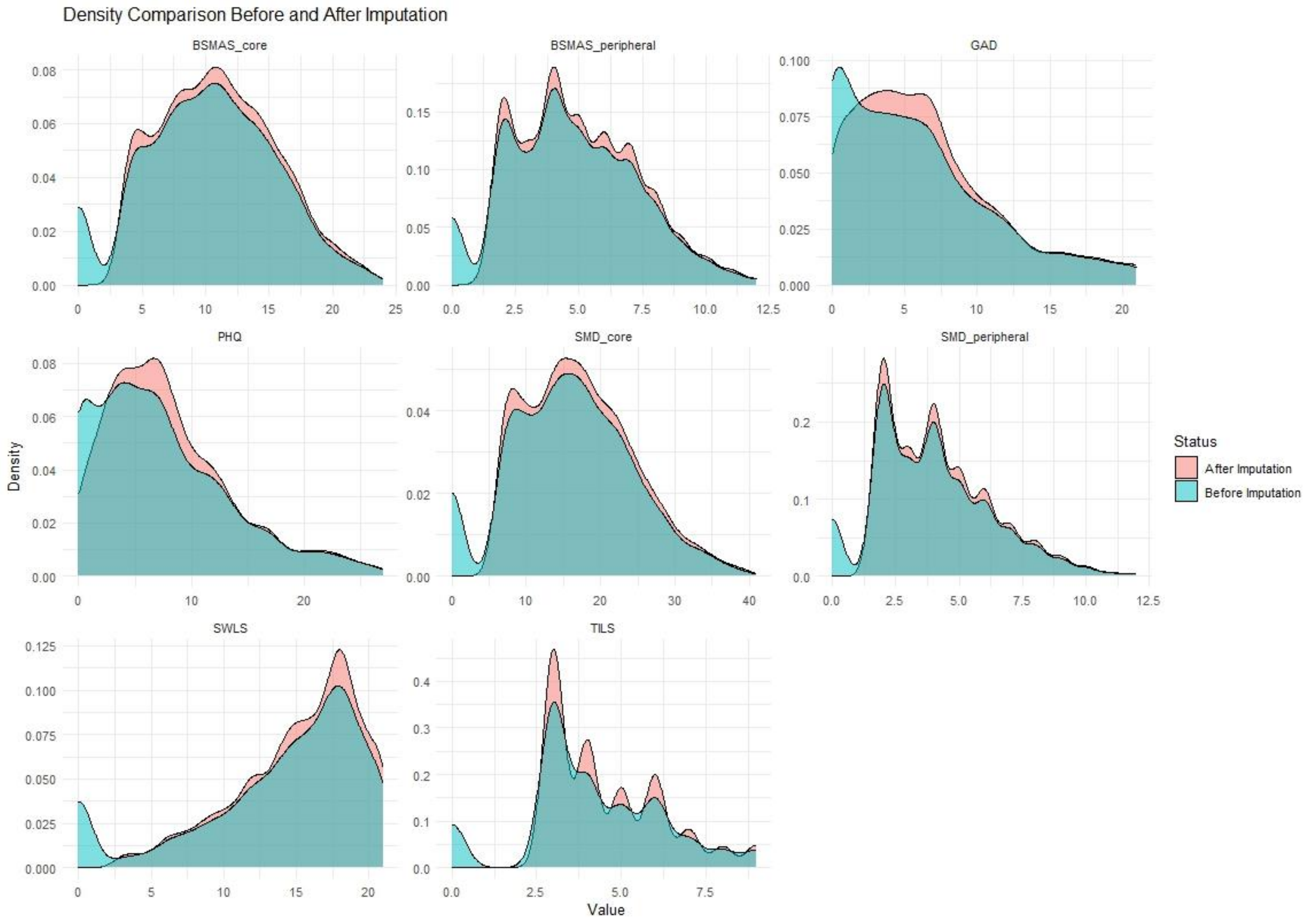

The Random Forest model yielded an out-of-bag error of .53. Given that the results were not optimal, analyses were repeated by using listwise deletion, which is detailed in the supplementary materials. The main conclusions of the study did not differ between the two approaches.

Table S2. Confirmatory factor analysis models based on listwise deletion (*N* = 2,323)

Model	χ^2	*df*	*p*-Value	CFI	TLI	RMSEA	SRMR	AIC	BIC	$\Delta\chi^2$	Δdf	*p*-Value comparison
SMD Model 1	477.925	27	<.001	.908	.878	.085	.049	66,375.14	66,478.65	-	-	
SMD Model 2	463.792	26	<.001	.911	.877	.085	.048	66,349.79	66,459.05	14.879	1	.003
SMD Model 3	417.855	26	<.001	.920	.890	.081	.045	66,285.29	66,394.55	54.183	1	<.001
BSMAS Model 1	162.910	9	<.001	.952	.921	.086	.037	44,784.93	44,853.94	-	-	
BSMAS Model 2	114.613	8	<.001	.967	.938	.076	.035	44,721.54	44,796.30	47.252	1	<.001
BSMAS Model 3	51.036	8	<.001	.987	.975	.048	.022	44,637.29	44,712.05	117.26	1	<.001

Note. SMD Model 1: one-factor model; SMD Model 2: two-factor model (tolerance, salience, and mood modification as peripheral criteria); SMD Model 3: two-factor model (tolerance and salience as peripheral criteria); BSMAS Model 1: one-factor model; BSMAS Model 2: two-factor model (tolerance, salience, and mood modification as peripheral criteria); BSMAS Model 3: two-factor model (tolerance and salience as peripheral criteria). The second and third models were evaluated against their nested model (the first model). To compare these nested models, we used the Satorra-Bentler scaled difference test.

Table S3. Internal consistency reliability estimates based on listwise deletion ($N = 2{,}323$)

Model	Factor	Cronbach's α	McDonald's ω
BSMAS Model 1	Factor 1	.828	.823
BSMAS Model 2	Factor 1	.712	.709
	Factor 2	.735	.755
BSMAS Model 3	Factor 1	.754	.753
	Factor 2	.769	.769
SMD Model 1	Factor 1	.860	.856
SMD Model 2	Factor 1	.809	.800
	Factor 2	.679	.707
SMD Model 3	Factor 1	.826	.819
	Factor 2	.707	.718

Note. BSMAS Model 1: one-factor model; BSMAS Model 2: two-factor model (tolerance, salience, and mood modification as peripheral criteria); BSMAS Model 3: two-factor model (tolerance and salience as peripheral criteria); SMD Model 1: one-factor model; SMD Model 2: two-factor model (tolerance, salience, and mood modification as peripheral criteria); SMD Model 3: two-factor model (tolerance and salience as peripheral criteria).

Figure S3. Standardized regression estimates for the two-factor SEM models of the BSMAS and the SMD based on listwise deletion (*N* = 2,323).

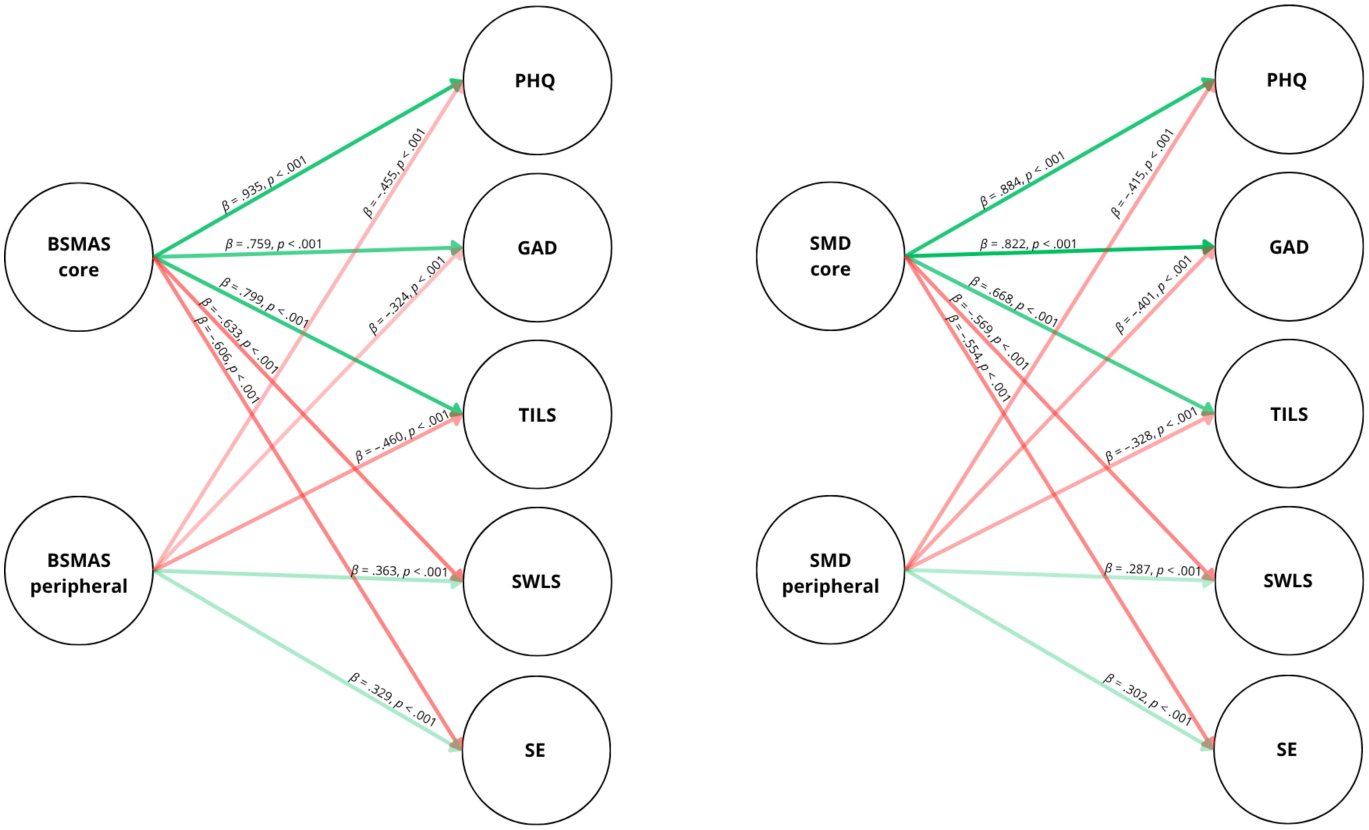

Note. For the sake of clarity, indicators, intercepts, covariances, thresholds, and unique variances are not depicted. To compute the SEM models, we selected the two-factor model for the two questionnaires (i.e., including only tolerance and salience as peripheral components).

Table S4. Factor loadings for core and peripheral criteria in the BSMAS and SMD based on listwise deletion (N = 2,323)

Scale	Item	Factor	Factor loadings (λ)	p-value
BSMAS	1	Peripheral	0.733	–
	2	Peripheral	0.861	< .001
	3	Core	0.775	–
	4	Core	0.569	< .001
	5	Core	0.614	< .001
	6	Core	0.648	< .001
SMD	1	Peripheral	0.723	–
	2	Peripheral	0.770	< .001
	3	Core	0.623	–
	4	Core	0.576	< .001
	5	Core	0.575	< .001
	6	Core	0.606	< .001
	7	Core	0.593	< .001
	8	Core	0.785	< .001
	9	Core	0.645	< .001

Note. Factor loadings for the first indicator of each factor were fixed to 1.00 for identification and are not tested.

Table S5. Fit indices for the two-factor SEM models of the BSMAS and the SMD based on listwise deletion (N = 2,323)

Model	χ^2	*df*	*p*-Value	CFI	TLI	RMSEA	SRMR
BSMAS	2,970.068	357	<.001	.945	.937	.056	.041
SMD	3,243.854	444	<.001	.940	.933	.052	.042

Table S6. Means, standard deviations, and correlations based on listwise deletion (*N* = 2,323)

Variable	*M*	*SD*	1	2	3	4	5	6	7	8	9	10
1. BSMAS	14.97	7.27										
2. Core BSMAS	10.27	5.18	.97**									
3. Peripheral BSMAS	4.70	2.56	.88**	.74**								
4. SMD	19.83	10.09	.83**	.82**	.70**							
5. Core SMD	15.92	8.26	.82**	.82**	.67**	.99**						
6. Peripheral SMD	3.91	2.31	.72**	.67**	.68**	.84**	.75**					
7. PHQ	7.37	6.06	.49**	.51**	.37**	.52**	.52**	.38*				
8. GAD	5.89	5.20	.46**	.46**	.36**	.48**	.48**	.35**	.81**			
9. TILS	4.19	2.10	.47**	.48**	.37**	.50**	.50**	.39**	.63**	.60**		
10. SWLS	13.87	5.93	-.24**	-.25**	-.14**	-.25**	-.26**	-.15**	-.52**	-.43**	-.45**	
11. SE	3.43	1.18	-.24**	-.26**	-.15**	-.24**	-.25**	-.14**	-.49**	-.42**	-.45**	.45**

Note. M and *SD* are used to represent mean and standard deviation, respectively. *$p < .05$. **$p < .01$.
